# Sintering of Lead-Free Piezoelectric Sodium Potassium Niobate Ceramics

**DOI:** 10.3390/ma8125449

**Published:** 2015-12-01

**Authors:** Barbara Malič, Jurij Koruza, Jitka Hreščak, Janez Bernard, Ke Wang, John G. Fisher, Andreja Benčan

**Affiliations:** 1 Jožef Stefan Institute, Jamova cesta 39, Ljubljana 1000, Slovenia; jitka.hrescak@ijs.si (J.H.); andreja.bencan@ijs.si (A.B.); 2 Jožef Stefan International Postgraduate School, Jamova cesta 39, Ljubljana 1000, Slovenia; 3 Technische Universität Darmstadt, Alarich-Weiss-Str. 2, Darmstadt 64287, Germany; koruza@ceramics.tu-darmstadt.de; 4 Slovenian National Building and Civil Engineering Institute, Dimičeva 12, Ljubljana 1000, Slovenia; janez.bernard@zag.si; 5 State Key Laboratory of New Ceramics and Fine Processing, School of Materials Science and Engineering, Tsinghua University, Beijing 100084, China; wang-ke@tsinghua.edu.cn; 6 School of Materials Science and Engineering, Chonnam National University, Gwangju 500-757, Korea; johnfisher@jnu.ac.kr

**Keywords:** lead-free piezoelectric, KNN, sodium potassium niobate, sintering, microstructure

## Abstract

The potassium sodium niobate, K_0.5_Na_0.5_NbO_3_, solid solution (KNN) is considered as one of the most promising, environment-friendly, lead-free candidates to replace highly efficient, lead-based piezoelectrics. Since the first reports of KNN, it has been recognized that obtaining phase-pure materials with a high density and a uniform, fine-grained microstructure is a major challenge. For this reason the present paper reviews the different methods for consolidating KNN ceramics. The difficulties involved in the solid-state synthesis of KNN powder, *i.e.*, obtaining phase purity, the stoichiometry of the perovskite phase, and the chemical homogeneity, are discussed. The solid-state sintering of stoichiometric KNN is characterized by poor densification and an extremely narrow sintering-temperature range, which is close to the solidus temperature. A study of the initial sintering stage revealed that coarsening of the microstructure without densification contributes to a reduction of the driving force for sintering. The influences of the (K + Na)/Nb molar ratio, the presence of a liquid phase, chemical modifications (doping, complex solid solutions) and different atmospheres (*i.e.*, defect chemistry) on the sintering are discussed. Special sintering techniques, such as pressure-assisted sintering and spark-plasma sintering, can be effective methods for enhancing the density of KNN ceramics. The sintering behavior of KNN is compared to that of a representative piezoelectric lead zirconate titanate (PZT).

## 1. Introduction

Piezoelectrics are an important group of functional materials with a wide range of applications, including sensors, actuators and transducer devices [1]. The most widely used materials for these applications are lead-based perovskites, predominantly Pb(Zr,Ti)O_3_ (PZT)-based ceramics, with excellent electromechanical properties. Along with the widespread research and industrial use, the processing methods for the preparation of these materials have been studied and optimized, see for example [2].

However, increased environmental and health concerns have triggered the search for alternative materials that could replace these lead-containing ceramics and result in less-hazardous production methods, use and recycling. This effort was additionally supported by governmental regulations with increasingly stringent demands [3,4,5]. Consequently, the research into novel, lead-free materials has expanded tremendously over the past 15 years and, currently, three main groups of materials are being considered: K_0.5_Na_0.5_NbO_3_ (KNN)-based, BaTiO_3_ (BT)-based, and Bi_1/2_Na_1/2_TiO_3_ (BNT)-based piezoelectrics. Although none of these materials can fully replace PZT in all applications, many compositions exhibit comparable or even better properties for specific requirements. For an overview of the development and current status of lead-free piezoelectrics, the reader is referred to ref. [6,7,8,9].

KNN-based compositions have been the most widely studied among the above-listed lead-free materials, as is evidenced by their approximately 50% share of the total number of publications in the field [9,10]. Although these materials were studied as early as the 1950s [11,12,13], intensive research only began after 2004 with the discovery of a large piezoelectric response in Li-, Ta-, Sb-modified-and-textured KNN [14]. Subsequent studies focused predominantly on investigations of the strain mechanisms, the crystallographic and domain structure, and an enhancement of the piezoelectric properties. The findings have been summarized in several reviews [6,7,8,10,15,16,17,18]. The main advantages of KNN over other lead-free compositions are the good temperature stability of the piezoelectric properties [19,20], the high mechanical quality factors [21,22], the fatigue resistance [23,24], the low density [25], the biocompatibility [26], and, as shown recently, the compatibility with low-cost, base-metal electrodes [27]. In addition to the scientific community, these materials have also attracted the attention of industry and the first prototypes and products started to appear: ultrasonic transducers [28], ultrasonic motors [29], multilayer actuators [30], knock sensors [31], and piezoelectric transformers [32].

Despite their promising properties, KNN-based piezoelectrics are still not widely used in industrial products. The frequently reported drawbacks are related to processing: difficulties in obtaining high densities of sintered products, deviations from the stoichiometry and the subsequent formation of secondary phases, and difficulties in controlling the microstructure, see for example [15,17,25,33,34,35]. Such phenomena can lead to inhomogeneous distributions of the applied electric fields, leakage currents, low breakdown fields, poor reproducibility and low piezoelectric performance of the ceramics, and should therefore be carefully addressed. While less attention was devoted to these problems in the initial research period of lead-free materials, several underlying mechanisms were revealed during the past decade. At this point it should be remembered that understanding the processing of PZT ceramics required several decades after their discovery in 1954, e.g., studies on stoichiometry control [36,37,38,39].

The aim of this study was to draw together the existing knowledge on various topics related to the sintering of KNN-based piezoceramics and so motivate further research in this field. The Introduction section is followed by a brief description of sintering theory and the relevant mechanisms for the densification and grain growth of polycrystalline ceramics (Section 2). Section 3 represents the main part of this work and is subdivided as follows: the first two parts introduce the KNN system, along with the phase relations and synthesis reactions (Section 3.1 and Section 3.2), while the third part focuses on the solid-state sintering, being the most widely used sintering technique (Section 3.3). This is followed by a review of liquid-phase sintering (Section 3.4), the influence of the sintering atmosphere on the microstructure (Section 3.5), and the enhancement of the densification by chemical modifications (Section 3.6) and alternative sintering techniques (Section 3.7). The special case of controlled abnormal grain growth and its use for the growth of single crystals is presented in Section 3.8. Finally, Section 3.9 compares the sintering of KNN materials to that of the well-established PZT materials.

## 2. Sintering of Ceramic Materials—Fundamentals

This section provides a brief description of the basic processes occurring during sintering, while for more details the reader is referred to [40,41,42,43].

Sintering is one of the oldest processing technologies and is by far the most widely used technique for fabricating many ceramic components. It can be described as an irreversible thermodynamic process in which powder compacts are consolidated using thermal energy to obtain dense, polycrystalline ceramics. The main driving force is the reduction of the surface free energy of an assembly of particles; this driving force can be increased by decreasing the particle size. Then, in addition to the curvature of the particle surface, which increases with decreasing particle size, contributions to the driving force include the application of external pressure or a chemical reaction [40].

The sintering process is an interplay between densification and grain growth, and is divided into three overlapping ***sintering stages***, depending on the changes in the grain size and shape, the pore size and shape, and the related densification kinetics. During the initial stage, the particles become connected with necks, their surface roughness decreases, and a porous network of interconnected particles with a relative density of 60%–70% is formed. The intermediate stage is characterized by rapid shrinkage due to the increased densification rate. The pores gradually diminish and the connections between them disappear, resulting in the “closed-porosity” state (usually at a relative density of 92%–95%). In the final stage, the densification rate dramatically decreases, while the isolated pores can either shrink and disappear or merge, and thus grow. The grain growth, which typically occurs during this stage, to a large extent depends on the grain-boundary mobility and the pore/grain-boundary interaction. In addition, abnormal grain growth can occur, resulting in an increase in the diffusion paths between the pores and the grain boundaries, and the entrapment of these pores within the large grains. These effects impede the material transport towards the pores and, consequently, the densification is halted.

**Figure 1:**
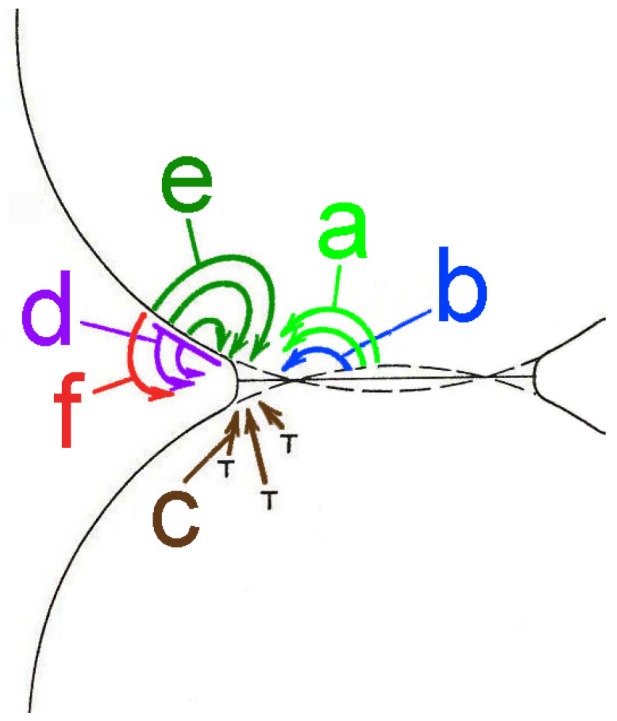
Material-transport mechanisms during solid-state sintering: lattice diffusion from the grain boundary (**a**) and from the surface (**e**) to the neck; grain-boundary diffusion (**b**); viscous flow (**c**); surface diffusion (**d**); and evaporation/condensation (**f**). The symbol “┬” marks the dislocations and other defects. (Reprinted with permission from [42]. Copyright 1976 Wiley-VCH.)

The processes occurring during solid-state sintering require ***material transport*** across the microstructure, which can take place via lattice diffusion, grain-boundary diffusion, surface diffusion, evaporation/condensation or viscous flow (Figure 1). While all these mechanisms promote neck growth, only some contribute to the densification (Figure 1a–c). The latter are, subsequently, referred to as the densifying mechanisms, while the others (Figure 1d–f) are known as the non-densifying mechanisms. It should be noted that the material transport can be further enhanced by the addition of a liquid phase, which will be discussed in more detail in Section 3.4.

The ***material-transport path*** via diffusion is determined by the different types of defects present in a polycrystalline material. As such, the activation energy for surface diffusion (*Q_S_*) is expected to be the lowest. Alternatively, the atoms can diffuse along the grain boundaries, which are regions of lattice mismatch and disorder between adjacent grains formed during the sintering process. The activation energy for the grain-boundary diffusion (*Q_GB_*) is expected to be as high as *Q_S_.* Moreover, the diffusion can take place via lattice point defects, either vacancies or interstitials, whereby the flux of vacancies should be compensated by the opposite and equal flux of atoms. This mechanism typically has the highest activation energy (*Q_L_*) and is therefore active at higher temperatures. However, the above-described relationships between the activation energies should be considered with care, as revealed by many experimental studies [41]. In addition, the fraction of free surfaces, grain boundaries and lattice atoms changes during sintering, thereby modifying the contributions of different mechanisms. It should also be noted that the diffusing species in ceramics are ions with different and opposite charges (ambipolar diffusion), often also with different diffusion rates, and therefore the preservation of the stoichiometry and electronegativity have to be taken into account. Furthermore, each ion may have more than one diffusion path and the diffusion rate is controlled by the slowest-diffusing ion along its fastest path. Besides the diffusion mechanisms described above, mass transport during sintering can also occur via evaporation/condensation. Here, the material transfer is promoted by the vapor-pressure difference between the positive curvature at the surface of the particle and the negative radius of the neck region. According to Kelvin´s Equation, the vapor pressure, and thus the material transport via evaporation/condensation, exponentially increases with the increasing curvature of the particles [42].

**Figure 2:**
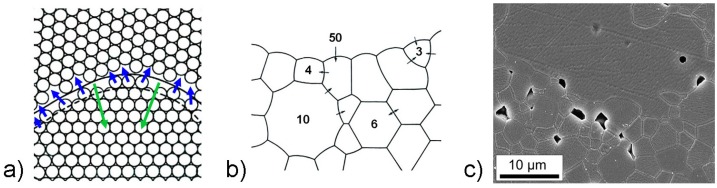
(**a**) Movement of the grain boundary towards its center of curvature (green arrows) by the diffusion of atoms across the boundary (blue arrows) (Reprinted with permission from [40]. Copyright 2003 CRC PRESS LLC). (**b**) Movements of grain boundaries (black arrows) depending on the number of sides in a polycrystalline ceramic (Reprinted with permission from [44]. Copyright 1990 Springer). (**c**) Grains with different sides and curvatures in NaNbO_3_, sintered for 15 min at 1350 °C (SEM image) (Reprinted with permission from [45]. Copyright 2011 The American Ceramic Society).

The process of ***grain growth*** typically takes place during the final sintering stage. If the average grain size of the material increases continuously and without changes in the grain-size distribution, the grain growth is referred to as normal. During this process the atoms diffuse from one side of the grain boundary to a new position on the other side and the driving force is the free-energy difference across the curved grain boundary. As a result, the grain boundary will move towards its center of curvature (schematically presented in Figure 2a). If the grain-boundary energy is isotropic, the balance of the grain-boundary tensions requires that the edges meet at 120° and, consequently, a hexagonal grain will have straight sides. On the other hand, the boundaries of the polygons with more than six sides will be concave, while in the polygons with fewer than six sides the boundaries will be convex. As grain boundaries migrate towards their centers of curvature, the grains with fewer than six sides tend to shrink, while the ones with more than six sides tend to grow (Figure 2b,c) [44].

In some materials, a small fraction of the grains can grow unusually quickly to reach a large size in a matrix of fine grains, which grow with a slow rate. This phenomenon is referred to as abnormal grain growth and results in a bimodal grain-size distribution, which is usually undesired. The causes of abnormal grain growth are generally related to specific local conditions, such as anisotropic grain-boundary energies and grain-boundary structures [46,47], the preferential segregation of dopants/impurities [48], and second-phase particles or pores, all of which affect the grain-boundary mobility. In addition, the presence of a liquid phase or a non-uniform particle/grain-size distribution is also often stated as a cause of the abnormal grain growth [40,49].

**Figure 3:**
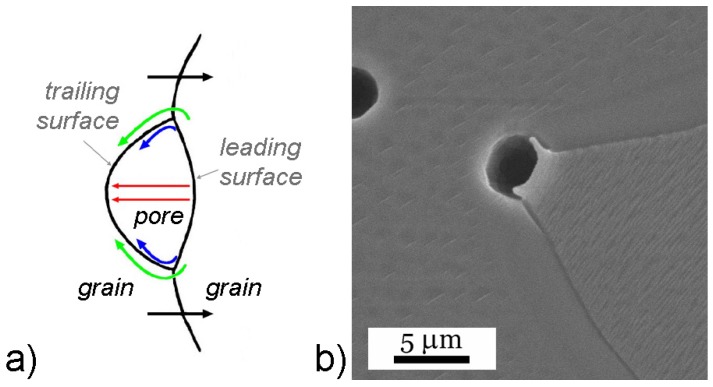
(**a**) Schematics of a pore moving with the grain boundary and possible material-transport mechanisms (blue: surface diffusion, green: lattice diffusion, and red: evaporation/condensation). (Reprinted with permission from [40]. Copyright 2003 CRC PRESS LLC). (**b**) A pore moving along with the grain boundary at the moment of separation from the boundary (NaNbO_3_ sintered for 2 h at 1350 °C; scanning electron microscopy (SEM) image) (Reprinted with permission from [45]. Copyright 2011 The American Ceramic Society).

The grain-boundary mobility is additionally affected by various factors, the most important of them being the pores. A grain boundary moving under the influence of its curvature applies a force on a pore situated at the boundary, resulting in a change of the pore shape (Figure 3) [50]. The chemical potential difference driving the atom flux is created by the different curvatures of the leading and trailing surfaces of the pore. The material transport can occur via surface diffusion, lattice diffusion or evaporation/condensation. The grain-boundary migration is determined by the balance between the number of pores, their mobility, and the intrinsic grain-boundary mobility [51,52]. When the grain-boundary-migration velocity exceeds the pore-migration velocity, pore/boundary separation occurs (Figure 3b). The entrapped pores are difficult to eliminate due to the long diffusion distances; therefore, these conditions typically represent the limit of densification during sintering [52].

## 3. Sodium Potassium Niobate System

### 3.1. Phase Relations

**Figure 4:**
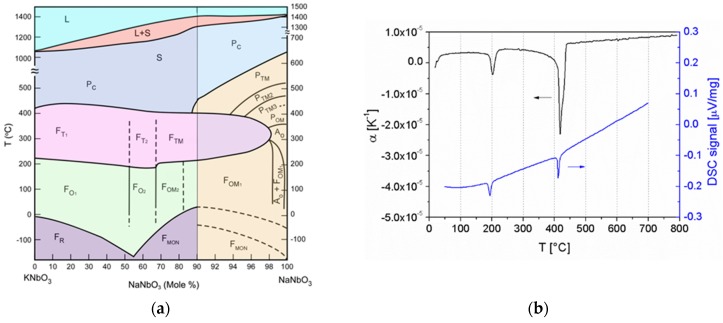
(**a**) Phase diagram of the KNbO_3_-NaNbO_3_ system (Reprinted with permission from [59]. Copyright 2006 The American Ceramic Society). (**b**) Phase transitions of the K_0.5_Na_0.5_NbO_3_ solid solution, as determined by dilatometry and differential scanning calorimetry upon heating (Reprinted with permission from [57]. Copyright 2011 The American Ceramic Society).

K_1-*x*_Na_*x*_NbO_3_ system is a pseudo-binary solid solution between the ferroelectric KNbO_3_ and the antiferroelectric NaNbO_3_. (Figure 4a) [18,25]. All the phases crystalize in the perovskite structure, but with different symmetries. While the KNbO_3_ was reported to have three temperature-induced ferroelectric transitions, the polymorphism of NaNbO_3_ is much more complex [53,54,55]. The phase transitions of the KNN solid solution with a K/Na ratio of 1/1 (KNN) are: rhombohedral $\overset{\sim 160℃}{↔}\;$orthorhombic $\overset{200℃}{↔}\;$tetragonal $\overset{410℃}{↔}\;$cubic phase (Figure 4b) [18,25,56,57]. The solidus and liquidus lines for this composition are at 1140 and 1420 °C, respectively [25]. Early studies suggested the coexistence of two different orthorhombic phases (O_1_ and O_2_) at a K/Na ratio of 1/1, forming a morphotropic phase boundary (MPB) and contributing to the enhanced piezoelectric properties [58]. However, later work revealed the absence of any symmetry changes in that region [56].

The room-temperature polymorph of KNN is often described as orthorhombic with the space group *Amm*2. However, strictly speaking, the perovskite-type ABO_3_ primary cell possesses a monoclinic symmetry [58,59]. The monoclinic primary cell features the lattice parameters *a*_m_ = *c*_m_ > *b*_m_, while the *b*_m_ axis is perpendicular to the *a*_m_*c*_m_ plane and its inter-axial angle β is slightly larger than 90° [60]. Hence, the perovskite-type primary cell of the KNN is monoclinic, while its unit cell has an orthorhombic symmetry at room temperature.

The phase-transition temperatures of KNN materials can be shifted by doping or the formation of pseudo-binary solid solutions. These modifications mostly result in a downward shift of the orthorhombic–tetragonal phase-transition temperature (*T*_O–T_) [19,61,62,63,64,65,66]. If the *T*_O–T_ is shifted to room temperature, the piezoelectric performance can be enhanced; this is known as the polymorphic phase-transition (PPT) effect [67]. Recently, it was found that KNN-based ceramics modified by BaZrO_3_, (Bi,Na,K,)ZrO_3_, (Bi,Li)TiO_3_, (Bi,Na)TiO_3_, *etc.*, possess a morphotropic phase boundary (MPB) that separates the tetragonal and rhombohedral phases, resembling that in classical lead-containing piezoelectrics [20,68,69].

### 3.2. Solid-State Synthesis of Powders

The most commonly used solid-state synthesis of KNN involves the reaction of alkali carbonates and niobium oxide [13], although other alkali compounds, such as nitrates [70], hydrogen carbonates [71] or sodium potassium tartrate hydrate [72], have been reported. The solid solution can also be formed by a reaction between the two end-member perovskites [56,73,74], often referred to as the perovskite route.

The summary reaction of alkali carbonates and niobium oxide is described by Equation (1), and according to thermogravimetric analyses it occurs between 400 and 700 °C [75]. The usually reported calcination temperatures are between 750 and 950 °C.

K_2_CO_3_(s) + Na_2_CO_3_(s) + 2 Nb_2_O_5_(s) → 4 K_0.5_Na_0.5_NbO_3_(s) + 2 CO_2_(g) (1)

Studies of the sodium or potassium oxide/carbonate–niobium oxide binaries revealed a number of phases with different alkali/Nb molar ratios [76,77,78,79,80], thus other phases besides the perovskite can be expected to form in the reaction(s) between K_2_CO_3_, Na_2_CO_3_ and Nb_2_O_5_. A diffusion-couples study of the ternary system (Na_2_CO_3_ + K_2_CO_3_)/Nb_2_O_5_ at 600 °C revealed that the reaction proceeded via the coupled diffusion of alkaline and oxygen ions into niobium oxide, and that the perovskite phase formed via an intermediate alkali polyniobate phase, (K,Na)_2_Nb_4_O_11_. The diffusion rate of the potassium ions was about one order of magnitude lower than that of the sodium ions, meaning that the reaction rate in the ternary system was determined by the diffusion of the slower potassium ions [81]. Clearly, as in any diffusion-controlled reaction, reducing the particle size should contribute to shorter diffusion paths and a lower thermal budget to complete the reaction [42].

**Figure 5:**
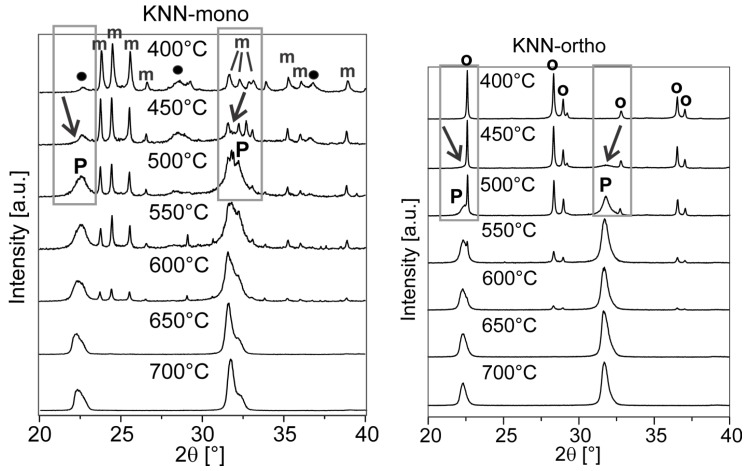
XRD patterns of the homogenized mixtures of KNN prepared from monoclinic and orthorhombic Nb_2_O_5_ precursors, denoted as KNN-mono and KNN-ortho, respectively, annealed at 400–700 °C. **o** stands for orthorhombic Nb_2_O_5_; **P** for the perovskite phase; **m** for monoclinic Nb_2_O_5_; and • for the poorly crystalline orthorhombic phase. The arrows mark the appearing perovskite phase. (Reprinted with permission from [82]. Copyright 2013 Elsevier).

Recently, it has been reported that the polymorphic form of niobium oxide strongly influences the course of the solid-state reaction [82]. Hreščak *et al*. prepared two batches of KNN using Nb_2_O_5_ powders in different polymorphic forms, one was orthorhombic and the other was monoclinic. Both Nb_2_O_5_ precursors were planetary milled before the homogenization step, so that uniform and comparable particle size distributions could be achieved. However, the X-ray diffraction (XRD) and transmission electron microscopy (TEM) analyses showed that after the planetary-milling step, the monoclinic Nb_2_O_5_ partly degraded into the orthorhombic polymorph and orthorhombic nanoparticles were attached to the surface of the micron-sized monoclinic particles. After a double calcination at 800 °C with intermediate and final planetary milling, the batch using the orthorhombic Nb_2_O_5_ yielded a homogenous single-phase KNN solid solution, while the batch using the originally monoclinic Nb_2_O_5_ reacted to form an inhomogeneous mixture of perovskite solid solutions containing significantly different molar ratios of K:Na (Figure 5). The latter could be homogenized by annealing the powder at 950 °C for 4 h, but sintering this powder resulted in a very poor density.

Alkali deficiency has been a frequently reported problem related to the synthesis of KNN. Such a deficiency could stem from the strong hygroscopicity of the potassium carbonate and result in the formation of polyniobate phases, some of which are hygroscopic even at room temperature [83]. Drying the carbonates at a minimum of 200 °C prior to any use and their manipulation in an inert atmosphere (dry-box) should be applied to avoid any possible deviations from stoichiometry, see for example Hagh *et al*. [73]. In order to avoid the alkali deficiency in KNN, a slight excess of alkali compounds was introduced in the reagent mixture even in early studies [13]. Alkali excess in the amount of a few mole % resulted in KNN particles with larger sizes than the stoichiometric or alkali-deficient KNN synthesized under the same processing conditions, *cf.* ref [33,84,85]. Recently, Haugen *et al*., hypothesized that alkali-containing oxides, such as KNN, will react with humidity and carbon dioxide to form surface carbonates and hydroxides that will create a liquid phase upon heating and promote the coarsening of particles. Such an effect would be even more pronounced in the case of fine particles with a high surface area [85].

**Figure 6:**
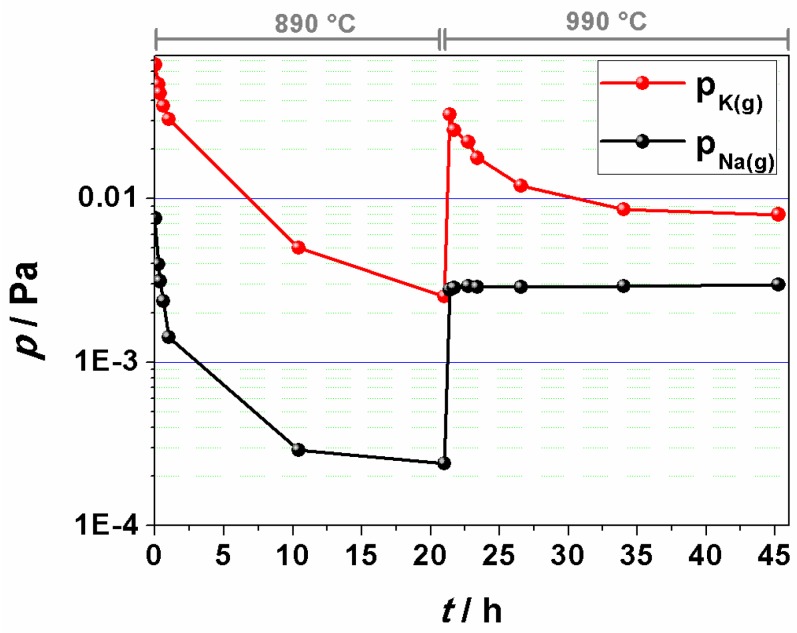
Time-temperature dependence of sodium and potassium vapor pressures over KNN, previously prepared by the calcination of the stoichiometric oxide-carbonate mixture at 950 °C for 12 h. Note that the analysis was performed in vacuum, so the results cannot be directly correlated with heating experiments performed in air, where the evaporation rates of the alkalis are much lower. (Reprinted with permission from [86]. Copyright 2015 The Royal Society of Chemistry).

Another problem related to KNN is assumed to be the high vapor pressure of alkalis at high temperatures, which might also result in an alkali deficiency in KNN. However, previous reports yielded contradictory results about which alkali is evaporating and to what extent. A recent Knudsen-effusion mass-spectrometry study revealed that the equilibrium vapor pressure of potassium over KNN is higher than that of sodium, *i.e.*, 8 × 10^−3^ Pa as compared to 3 × 10^−3^ Pa at 990 °C [86]. The KNN powder was, prior to the analysis, synthesized by heating a stoichiometric carbonate-oxide mixture at 950 °C for 12 h in an almost closed, platinum crucible in the air. The as-synthesized powder was not in thermodynamic equilibrium, meaning that potassium, and to a lesser extent also sodium, were not completely incorporated into the perovskite structure, as evidenced by the initially high and progressively decreasing vapor pressures of the two alkalis, see Figure 6. The equilibrium could be achieved only by additionally extended heating, for example, in the case of potassium, for 21 h at 890 °C and 23 h at 990 °C.

These findings suggest that the selected powder-synthesis conditions not only prevented the evaporation of alkalis upon heating but also contributed to a CO_2_-enriched atmosphere in the crucible, which could shift the equilibrium of the KNN formation reaction in the direction of the reactants, see Equation (1). Furthermore, it could be concluded that repeated calcination steps with intermediate milling, as reported by many groups, could indeed contribute to the completion of the solid-state reaction.

### 3.3. Solid-State Sintering of KNN

Poor densification has been reported as one of the main problems associated with alkaline-niobate-based piezoceramics, preventing their widespread application. Despite this, only a few systematic reports on the sintering of these materials can be found in the literature. The following sections summarize the existing knowledge about the processes occurring during the solid-state sintering of alkaline niobates, such as material-transport mechanisms, microstructure development, and grain growth.

#### 3.3.1. Sintering Mechanisms in NaNbO_3_

The investigation of sintering mechanisms in complex oxides, such as KNN, is difficult due to the ambipolar diffusion [81], the presence of two volatile oxides [86], the anisotropy of the surface energy [87,88], and the possible simultaneous activation of several diffusion mechanisms. It was therefore reasonable to investigate a less-complex model system, such as NaNbO_3_—the end-member of the KNN and several other alkaline niobate systems [6,8].

**Figure 7:**
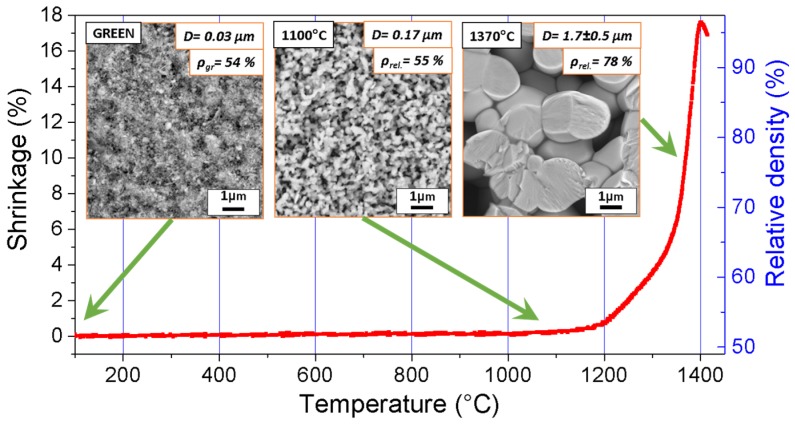
Dynamic sintering curve of the nano-sized NaNbO_3_ compacts while heating at 10 °C/min. The insets show the field-emission scanning electron microscopy (FE-SEM) images of the fracture surfaces of the green sample and the samples quenched from the marked temperatures (D-average grain size, ρ-relative density).

**Figure 8:**
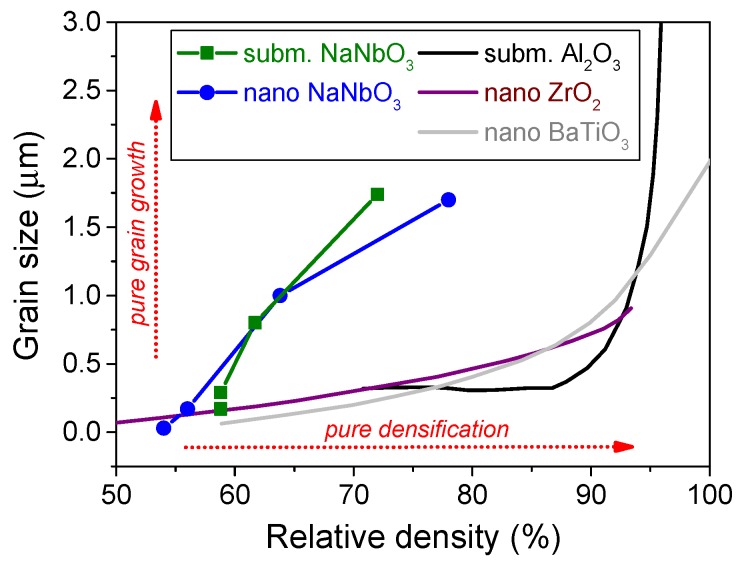
Microstructure-development trajectories for submicron- and nano-sized NaNbO_3_ [90], submicron-sized Al_2_O_3_ [94], nano-sized ZrO_2_ [95], and nano-sized BaTiO_3_ [96] powder compacts.

The sintering mechanisms in NaNbO_3_ were investigated using powders with two different particle sizes: a submicrometer-sized powder with a primary particle size of 100–300 nm (submicron-NaNbO_3_) was prepared by conventional solid-state synthesis [55], while the nanometer-sized powder with a particle size of about 25–30 nm (nano-NaNbO_3_) was obtained by top-down processing using a solid-state synthesis and colloidal milling [89]. The sintering behavior of both powders was studied using optical dilatometry and microstructure analysis, and the material-transport mechanisms were investigated [90]. The dynamic sintering curves revealed a very narrow shrinkage interval just below the melting point, as typically observed in alkaline niobates [91,92]. Although the densification of the nano-NaNbO_3_ started at a 100 °C lower temperature, both samples reached the same relative density at 1280 °C and their densification curves coincided at higher temperatures. The investigation of the microstructure development revealed no densification and considerable coarsening during the initial heating stage between room temperature and 1100 °C, since the grains of the submicron-NaNbO_3_ and the nano-NaNbO_3_ grew by 70% and 460%, respectively (Figure 7). Upon further heating both samples started to densify and reached a density of about 75% and a grain size of 1.7 μm at the highest investigated temperature of 1370 °C. Note that low-temperature coarsening was also previously reported for NaNbO_3_ powders prepared by other synthesis routes [93]. The competing effects of densification and grain growth during the heating of NaNbO_3_ are summarized in Figure 8 in the form of microstructure-development trajectories. The comparison reveals that regardless of the initial powder particle size, both NaNbO_3_ samples exhibit similar sintering behavior with considerable grain growth during the initial sintering stage, before the activation of the main densification process. It should be noted that this behavior is very different to that of other ceramic systems with comparable particle sizes, for which the trajectories are typically exponential functions with rapid densification and negligible grain growth during the intermediate sintering stage and considerable grain growth once a critical density is reached [94,95,96].

Rapid grain growth during the initial sintering stage and the absence of densification indicate the early activation of non-densifying material-transport mechanisms. The determination of the exact mechanism was performed by following the specific surface-area reduction during the isothermal sintering of nano-NaNbO_3_ powder compacts [90,97]. Surface diffusion was identified as the dominant sintering mechanism during the initial sintering stage of NaNbO_3_ and the corresponding activation energy was determined to be about 50–60 kJ/mol. For comparison, the corresponding value for Al_2_O_3_ is 536 kJ/mol [41]. The low activation energy of NaNbO_3_ results in the early activation of the surface diffusion and, consequently, grain growth during the initial stage, which explains the different behavior of NaNbO_3_ as compared to other ceramics. In other words, the grain growth occurring during the heating stage reduces the driving force for sintering before the sample reaches the main sintering stage and could therefore be identified as the main reason for the poor densification of the NaNbO_3_. Although the exact origin of the low activation energy for the surface diffusion remains unclear, it could be related to the low crystal-lattice energy of NaNbO_3_ [98], which was calculated to be more than one order of magnitude lower than for other perovskites and simple oxides [99,100]. To the best of our knowledge a study of the material-transport mechanisms in KNN has not been performed yet; however, due to the chemical similarity and low crystal-lattice energy of KNbO_3_ [98] the same mechanisms are expected to be active in other alkaline niobates.

#### 3.3.2. Solid-State Sintering of KNN: Stoichiometry and Microstructure

**Figure 9:**
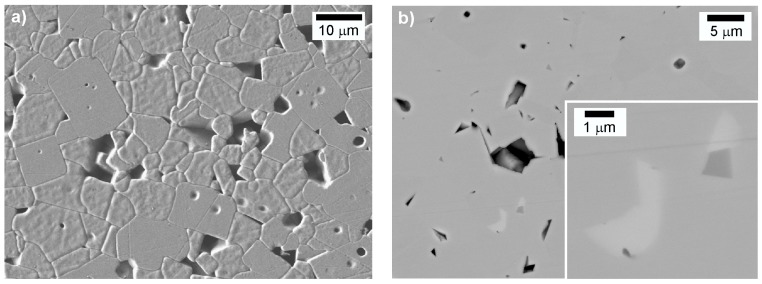
Microstructure of the KNN ceramic sintered at 1120 °C for 2 h, with a relative density of 92.4%, (**a**) thermally etched surface, (Reprinted with permission from [82]. Copyright 2013 Elsevier), SEM secondary electrons image (SEI), (**b**) polished surface, SEM backscattered electrons image (BEI), inset showing the white secondary tungsten-bronze type phase.

Stoichiometric KNN densifies in a narrow temperature range, most often only a few 10 °C below the solidus temperature at 1140 °C [75,82,91,101]. A typical microstructure of solid-state-sintered KNN consists of cuboidal grains (see Figure 9). A bimodal microstructure has frequently been reported, with intragranular pores within the large grains, for example in KNN, as a consequence of the abnormal grain growth (refer to Section 2).

Alkali deficiency results in the appearance of a secondary phase within the perovskite matrix. The secondary phase has the tungsten-bronze structure with the formula K_6_Nb_10.88_O_30_ (International Centre for Diffraction Data - ICDD: 87-1856) [101,102] or K_2_Nb_4_O_11_ [103].

**Figure 10:**
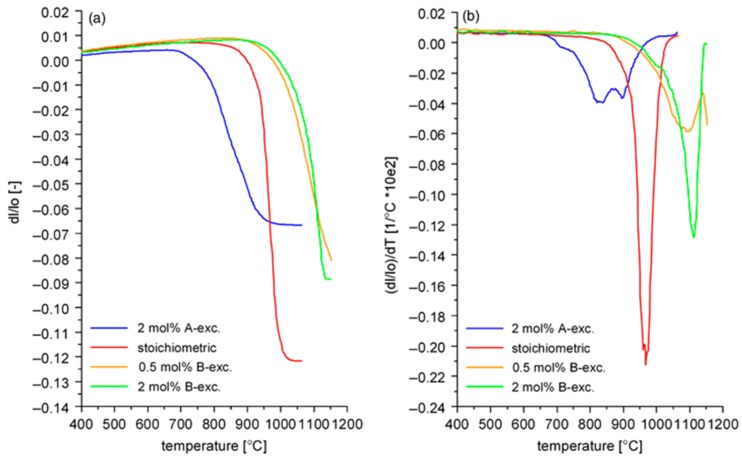
Sintering curves (**a**) and shrinkage rates (**b**) of KNN powder compacts with different stoichiometries (Reprinted with permission from [33]. Copyright 2010 The American Ceramic Society).

**Figure 11:**
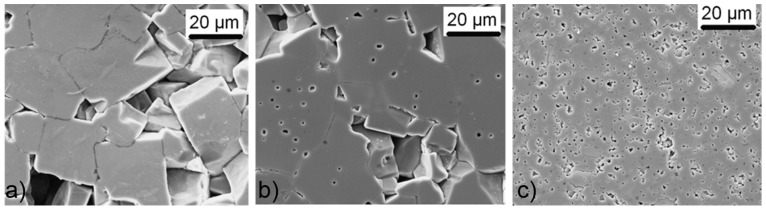
Microstructures of (**a**) alkali-excess (2%); (**b**) stoichiometric; and (**c**) Nb-excess (2%) KNN ceramics, sintered at 1105 °C, 2 h (Reprinted with permission from [33]. Copyright 2010 The American Ceramic Society).

Acker *et al*., studied the influence of stoichiometry on the sintering of KNN [33,104]. They compared the stoichiometric, alkali excess (2 mol %) and Nb-excess (0.5 and 2 mol %) formulations. The particle sizes of the powders, calcined at 775 °C for 5h, depended on the stoichiometry, as discussed in Section 3.2: the stoichiometric and Nb-excess powders were monomodal, with an about 0.6 μm particle size, while the alkali-excess powder had a bimodal size distribution, with peaks at 0.3 and ~3 μm. The stoichiometric KNN in this study exhibited the largest shrinkage with the typically narrow shrinkage interval although at lower temperatures than previously reported [91], while the alkali-excess KNN had the lowest shrinkage, but started to densify even below ~800 °C, see Figure 10. Upon sintering at 1105 °C for 2 h, the relative densities of the stoichiometric, alkali excess and Nb-excess (2 mol %) KNN were 95.3%, 86.5% and 90.9% of theoretical density (TD). The microstructure of the stoichiometric KNN was coarse-grained (about 10 μm) with intragranular porosity. Such a microstructure was the result of a period of abnormal grain growth at intermediate temperatures (1030 °C), as confirmed by the microstructural analysis, which also limited the possibility of achieving higher densities. The Nb-excess KNN consisted of finer grains than the stoichiometric compound. In the case of the alkali-excess KNN, considerable grain growth was already observed at intermediate temperatures, *i.e.*, ~5 μm at 970 °C for 5 min, which could be related not only to the early activation of the surface diffusion, which contributes to coarsening, but also to the presence of an intergranular liquid phase, as evidenced by SEM. The final microstructure consisted of coarse grains; however, without intragranular pores. The microstructures of the stoichiometric, alkali-excess and Nb-excess KNN are collected in Figure 11.

A common measure to reduce the loss of volatile species during sintering is to embed the powder compact into “atmospheric powder”, also referred to as packing or sacrificial powder. Early investigations of the PbZrO_3_-PbTiO_3_ system suggested reducing the loss of PbO by using atmospheric powder of the same composition or such with a higher PbO vapor pressure [38] (see also Section 3.9). The utilization of atmospheric powders has therefore also been suggested as a solution for the sintering of KNN. Most researchers used an atmospheric powder with the same composition (or with a slight alkali excess) as the sintered KNN sample. The success of this approach is still not clear: while some researchers reported increased densities [105], others observed only improvements to the grain size distribution, while the density decreased [106]. A systematic study of this problem was performed by Acker *et al.* [107]. The final grain size, morphology and density were found to be very sensitive to small variations in the stoichiometry of the sintered samples and the surrounding atmosphere. A density improvement using atmospheric powder could only be observed for the KNN samples with Nb-excess, while the density for the stoichiometric and alkali-excess KNN samples was reduced. The highest density of 4.34 g/cm^3^ (about 96% TD) was achieved for the stoichiometric KNN sample sintered without any atmospheric powder.

### 3.4. Liquid-Phase Sintering

Sintering in the presence of a liquid phase, known as liquid-phase sintering (LPS), has been recognized as an effective way of enhancing densification due to the formation of a liquid phase (sintering aid) in the solid matrix upon heating. The sintering aid should have a lower melting point than the matrix phase and it should be liquid (in the molten state) at the sintering temperature. Good wetting of the matrix, *i.e.*, a low contact angle between the liquid and the matrix phase, contributes to the efficient rearrangement and densification of the solid skeleton [108].

LPS has proved to be an effective way of preparing dense KNN ceramics, and in many studies the main purpose of the addition of a sintering aid was to increase the density and not so much to decrease the sintering temperature. Nevertheless, the latter could be achieved by a careful selection of sintering aids, and such an approach could contribute to reducing the expected losses of alkalis due to evaporation.

Various compounds have been proposed as LPS aids for KNN ceramics, including copper- and zinc-based compounds, e.g., CuO [109], K_4_CuNb_8_O_23_ [71,110], K_5.4_Cu_1.3_Ta_10_O_29_ [111], ZnO [112], and K_1.94_Zn_1.06_Ta_5.19_O_15_ [113], and borate or germanate-based glassy-phases, e.g., Na_2_B_4_O_7_∙10H_2_O (borax) [114] and K,Na–germanate [115,116]. The influences of selected sintering aids on the sintering temperature, density, Curie temperature and basic piezoelectric properties (*d*_33_, *k_p_*, *Q_m_*) of KNN are collected in Table 1.

The copper-based additives were studied the most thoroughly, as in addition to promoting densification, they also contributed to a hardening of the piezoelectric response of KNN (see also the next sections). The addition of 0.4 mol % CuO to alkali-deficient KNN or 0.5 mol % of K_4_CuNb_8_O_23_ to stoichiometric KNN resulted in ceramics with an almost theoretical density upon sintering at 1100 °C for 1 h. The latter sintering aid was eventually found as a reaction product in the KNN-CuO system. The abnormal grain growth of the KNN matrix, observed in both studied systems, could be explained by a too small amount of liquid phase and its non-homogeneous distribution in the solid matrix.

Effective lowering of the sintering temperature of KNN was achieved by using glassy sintering aids; however, at the expense of a less-effective densification. KNN with 0.15–0.90 wt % of Na_2_B_4_O_7_∙10H_2_O (melting point: 743 °C) sintered at 1020 °C for 4 h reached densities between 90% and 95.5% TD [114].

**Figure 12:**
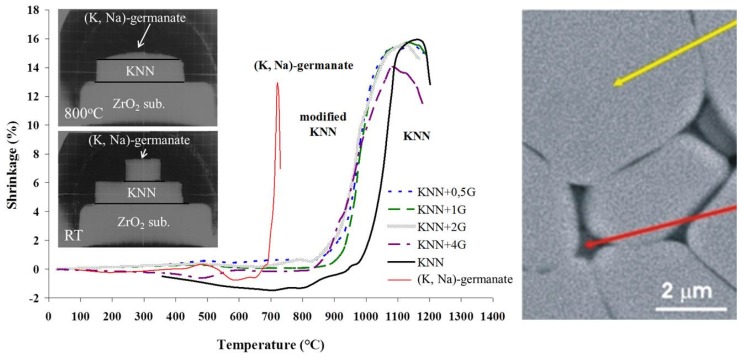
Shrinkage *versus* temperature of KNN, (K,Na)-germanate and KNN modified with 0.5, 1, 2 and 4 wt % of (K,Na)-germanate (KNN + 0,5G, KNN + 1G, KNN + 2G, KNN + 4G) powder compacts. Photos of the wetting experiments in a heating-stage microscope at room temperature and at 800 °C are given as insets. (Reprinted with permission from [115]. Copyright 2008 The American Ceramic Society.) Right: microstructure of the KNN + 4G sintered at 1000 °C for 8 h (SEM-BEI). The yellow and red arrows mark the KNN grain and the intergranular germanate phase, respectively. Note the good wetting of the matrix grains and the slightly rounded grain boundaries (Reprinted from [116] with the permission of the author).

The suitability of the (K,Na)-germanate (KNG, K/Na/Ge molar ratio = 1/1/2) amorphous phase as a sintering aid for KNN ceramics was evaluated on the basis of its wetting properties and from the shrinkage of the KNN-KNG green compacts during heating (Figure 12). KNG melts at around 720 °C, and at 800 °C it completely wets the surface of the KNN ceramic. Note the very low contact angle in Figure 12. Green compacts of KNN with different amounts of KNG started to densify between 850 and 900 °C, and achieved their maximum shrinkage at 1050 °C, which is almost 100 °C lower than for pure KNN. The good wetting of the KNN matrix grains with the KNG intergranular phase is also clearly seen in the microstructure, shown in Figure 12. Like with the previously discussed CuO-modified KNN, abnormal grain growth, resulting in a bimodal grain size distribution, was observed in KNN with a small amount of KNG (0.5 wt %), but it could be avoided for higher fractions of KNG (1–4 wt %). Nevertheless, the optimum piezoelectric response was obtained at 1 wt % of KNG [115,116].

**Table 1:** The effects of sintering aids on the Curie temperature, density and piezoelectric properties of the KNN ceramics.

Properties	KNN + 0.5 mol % K_4_CuNb_8_O_23_	KNN + 0.38 mol % K_5.4_Cu_1.3_Ta_10_O_29_	KNN + 0.5 mol % K_1.94_Zn_1.06_Ta_5.19_O_15_	KNN + 1.0 mol % ZnO	KNN + 1 wt % (K,Na)-Germanate	KNN + 0.45 wt % Na_2_B_4_O_7_∙10H_2_O
Sintering Temperature (°C)	1095	1125	1070	1050	1000	1020
Density (g/cm^3^, %)	4.40 (97.6)	4.60 *	≈4.35 (96.3)	4.25 *	4.31 (95.6)	≈95.5 *
Curie Temperature (°C)	410	≈390	390	≈400	≈400	-
*d*_33_	180 pm/V	190 pm/V	126 pC/N	123 pC/N	120 pC/N	132 pC/N
*k_p_* (/)	0.39	0.41	0.42	0.4	0.40	0.35
*Q_m_* (/)	1210	1300	≈60	≈140	77	109
Reference	[71]	[111]	[113]	[112]	[115]	[114]

* Only the absolute or relative density of the material is reported.

### 3.5. Controlled-Atmosphere Sintering of K_0.5_Na_0.5_NbO_3_-Based Ceramics

The majority of studies have used sintering in air, but for certain applications, sintering in a controlled atmosphere may be necessary. Multilayer actuators using 0.95K_0.5_Na_0.5_NbO_3_–0.05LiTaO_3_ ceramics and 70Ag-30Pd electrodes with reasonable performance ($d_{33}^{*}$ = 292 pm/V) have been developed [117]. Due to the high cost of Pd, the use of Ni electrodes would be an option to reduce production costs. Ni must be sintered in a reducing atmosphere and so the sintering behavior of KNN ceramics in different atmospheres is of interest. In addition, changing the sintering atmosphere will affect the vacancy concentrations in KNN, which will in turn affect the electrical properties.

Kawada *et al*. co-fired 0.96K_0.5_Na_0.5_NbO_3_-0.04CaZrO_3_ + 0.03ZrO_2_/Ni multilayer actuators at 1080 °C under an oxygen partial pressure ($P_{O_{2}}$) of 1 × 10^−11^ to 1 × 10^−10^ MPa [27]. The thickness of the ceramic layers was ~35 μm and the grain size was ~0.7 μm. For an electric field of 2 kV/mm, a value of $d_{33}^{*}$ = 360 pm/V was obtained. With the addition of 5 mol % LiF as a sintering aid, Kobayashi *et al.* were able to reduce the co-firing temperature of their KNN/Ni multilayer actuators to 1000 °C in an atmosphere of $P_{O_{2}}$ = 1 × 10^−11^ MPa [30]. A TEM analysis showed that there was no significant Ni diffusion into the ceramic and that the ceramic/electrode interface was sharp and free of secondary phases. Bulk ceramic samples fired in a reducing atmosphere also had a higher resistance and an improved DC degradation behaviour than air-fired ceramics, which was attributed to a reduction in the concentration of alkali metal vacancies. Liu *et al.* used a NaF-Nb_2_O_5_ liquid-phase sintering aid to facilitate the co-firing of a [K_0.5_Na_0.5_Nb_0.8_Ta_0.2_O_3_]/Ni multilayer actuator at 1150 °C under a reducing atmosphere of $P_{O_{2}}$ = 1 × 10^−11^ MPa. A re-oxidation heat-treatment at 850 °C under an atmosphere of $P_{O_{2}}$ = 1 × 10^−8^ MPa was then used to reduce the oxygen-vacancy concentration and increase the sample’s resistance by an order of magnitude [118].

Fisher *et al*., studied the effect of sintering atmosphere on the densification, structure and microstructure of KNN [119,120,121]. Sintering KNN in 75N_2_-25H_2_ (mol %) or H_2_ atmospheres caused an increase in the density relative to the samples sintered in air or O_2_ [121]. This increase in the density was attributed to an increase in the oxygen vacancies or a decrease in the critical driving force for the densification of materials with faceted grain boundaries [122]. A similar behavior was also noted by other workers for KNN [123] and NaNbO_3_ [124]. Sintering KNN in 75N_2_-25H_2_ (mol %) and H_2_ atmospheres caused a decrease in the orthorhombic distortion of the unit cell (calculated from XRD patterns) as well as a decrease in the orthorhombic–tetragonal and tetragonal–cubic phase-transition temperatures, as measured by differential scanning calorimetry, high-temperature XRD and high-temperature Raman scattering [120,121]. The phase transitions also deviated from first-order transitions towards diffuse transitions as the sintering atmosphere became more reducing. These changes were attributed to an increase in the oxygen-vacancy concentration and decreased A-site ordering.

**Figure 13:**
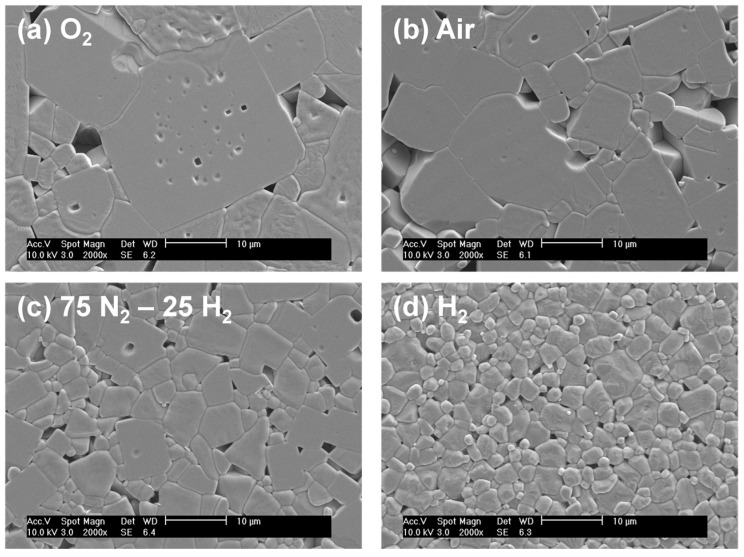
SEM micrographs of K_0.5_Na_0.5_NbO_3_ ceramics sintered at 1100 °C in different atmospheres (Reprinted with permission from [121]. Copyright 2010 Elsevier).

Sintering in reducing atmospheres also had significant effects on the microstructure [119,120,121]. The samples sintered in oxygen or air at 1100 °C showed an abnormal grain growth, while the samples sintered in 75N_2_-25H_2_ (mol %) or H_2_ atmospheres showed a transition from abnormal grain growth to normal grain growth (Figure 13). The changes in grain growth behavior were explained by considering the effect of the sintering atmosphere on the grain-boundary structure. KNN has grain boundaries that are faceted on an atomic scale [119]. In such a case, grain growth takes place by interfacial reaction-limited mechanisms, such as 2D nucleation and growth or step growth, rather than by diffusion-limited growth [125,126,127]. For interfacial reaction-limited grain growth, the type of grain growth behavior (pseudo-normal, abnormal or stagnant) depends strongly on the value of the step’s free energy, which is the excess energy of a step or 2D nucleus on the surface of a crystal. The step free energy can be reduced by increasing the configurational entropy of a material [128,129]. This can be increased by increasing the vacancy concentration [130]. Sintering in reducing atmospheres caused an increase in the concentration of oxygen vacancies, which in turn reduced the step’s free energy and caused a transition from abnormal to pseudo-normal grain growth.

The choice of sintering atmosphere can also affect the behavior of dopants added to KNN. Wang *et al*., sintered CuO-doped KNN ceramics in air and Ar atmospheres [131]. The air-sintered samples showed a characteristic double P-E hysteresis loop and a high value of the quality factor *Q_m_*, implying that the Cu^2+^ ions were acting as B-site acceptor dopants. The Ar-sintered samples only showed a slight pinching of the P-E loop and a lower value of *Q_m_*, implying that a relatively small number of Cu^2+^ ions were acting as acceptors. They suggested that in Ar, most of the Cu^2+^ ions are reduced to Cu^+^ and then substitute isovalently for Na^+^ on the A-site. As a result, the acceptor-doping effect is lower than for the air-sintered samples. Kobayashi *et al*., found that Sn could act as an acceptor or a donor dopant in SnO-doped KNN, depending on the sintering atmosphere [132]. For $P_{O_{2}}$ > 1 × 10^−11^ MPa, the Sn^4+^ substituted for Nb^5+^ as a B-site acceptor, whereas for $P_{O_{2}}$ < 1 × 10^−11^ MPa, the Sn^2+^ mainly substituted for Na^+^ or K^+^ as an A-site donor. Sintering in atmospheres with $P_{O_{2}}$ < 1 × 10^−11^ MPa resulted in a “softening” effect, with an increased *d*_33_ and a lower *E_C_*. This was due to the reduced concentration of domain wall-pinning $2Sn_{Nb}^{\prime }-V_{O}^{••}$ defect complexes. The conductivity was also reduced, as the Sn^2+^ donors compensated for the $V_{Na}^{\prime }$ and $V_{K}^{\prime }$ acceptor defects.

### 3.6. Chemical Modifications

The most versatile and relatively inexpensive way to improve the density of KNN is the chemical modification of KNN ceramics while using a conventional sintering technique. The following two sections summarize the research on doping and the formation of a solid solution for the KNN system and the related influence on sintering.

#### 3.6.1. Doping

The importance of lattice vacancies for the densification of perovskites was emphasized by Jaffe in his standard textbook [25]. If A-site vacancies are introduced, the diffusion is enhanced, while the oxygen vacancies act as diffusion inhibitors.

One of the earliest works investigating the influence of doping on KNN densification is the article from 1975 by Kosec and Kolar [91]. The authors reported an improvement in densification by using excess Nb^5+^ (B-site) ions and incorporating Mg^2+^ (A-site donor dopant) into the perovskite lattice. Both routes were suggested to produce A-site vacancies and therefore improve the material transport during sintering. Although undoped KNN did not sinter well, even for increased sintering times, the density of KNN with 1% Mg^2+^ could be improved up to 4.38 g/cm^3^ (97% TD) when sintered for 24 h at 1125 °C. The importance of the A-site vacancies was also demonstrated with an experiment, when excess A-sites (alkalines) were added to KNN and densities lower than those of the undoped KNN were obtained.

In 2005, Malič *et al.* [72] performed a study that experimentally demonstrated that A-site vacancies improve the sintering of perovskites, as was suggested by Jaffe [25]. They doped KNN with 0.5 at. % of the alkaline earth metals Mg^2+^, Ca^2+^, Sr^2+^ or Ba^2+^, since these elements should act as A-site donor dopants and therefore cause the formation of A-site vacancies in KNN and sintered the ceramics at 1115 °C for 2 h. Compared to the undoped KNN (ρ = 94.4% TD), doping with Sr^2+^ improved the density up to 96.0% TD. The other dopants did not prove to have a positive effect on the density of KNN: doping with Ba^2+^ resulted in 94.1% TD, but also in a secondary phase formation, while doping with Mg^2+^ resulted in 91.2% TD, and KNN doped with Ca^2+^ had a density of 93.8% TD.

Taub *et al.* [133] prepared KNN with 0, 0.5, 1 and 2 mol % of Ca^2+^ or Ba^2+^. The samples were prepared by solid-state synthesis and sintered at 1125 °C for 2 h. All the doped samples contained a secondary phase. Adding 0.5 mol % of Ba improved the density of the KNN up to 4.33 g/cm^3^, *i.e.*, 96.0% TD (compared to 4.28 g/cm^3^, *i.e.*, 94.9% TD for undoped KNN). The addition of higher concentrations of dopants than 0.5 mol % resulted in a drastic density decrease. The authors offered the following explanation: the Ca^2+^ and Ba^2+^ enter the A-site with the Ba^2+^ entering preferably the K^+^ position (ionic radii 135 and 138 pm, respectively) and Ca^2+^ replacing more likely the Na^+^ (ionic radii 100 and 102 pm, respectively). The segregation of K^+^ or Na^+^, respectively, would occur and modify the microstructure and phase composition, which would then influence the density.

Vendrell *et al.* [134] prepared KNN doped with 0.5 at. % Zr^4+^ or 0.5 at % Ti^4+^. According to the charge compensation mechanism, the oxygen vacancies should be formed in the perovskite lattice, as is probable for a B-site acceptor dopant. The assumption of oxygen-vacancy formation was supported by the other measurements. Although the presence of the oxygen vacancies in perovskites was previously suggested by Jaffe [25] to hinder the densification, the KNN with Zr had 98.2% TD and the KNN doped with Ti had 97.4% TD, compared to the 95.1% TD achieved for the undoped KNN (see also Section 3.5).

Zuo and Rödel [135] studied the densification of KNN doped with different oxides, which was first calcined and then attrition milled together with sintering aids in the form of different oxides. The attrition milling itself resulted in a significant improvement to the density of the undoped KNN, *i.e.*, as high as 98.4% TD for the sample milled for 48 h, while 6 h of attrition milling helped to achieve undoped KNN with a density of 96.0% TD. The addition of 1 mol % of ZnO resulted in 97.5% TD, and 1 mol % of SnO_2_ addition resulted in a density of 98.6% TD, both attrition milled for 6 h and sintered at 1100 °C for 4 h. CdO and Sc_2_O_3_ did not have a significant influence on the final density of the K_0.5_Na_0.5_NbO_3_, while CeO_2_, Y_2_O_3_, WO_3_ severely inhibited the densification.

One of the best-studied sintering-aids is that of Cu-based sintering aids (refer also to the previous sections). Matsubara [109] studied the influence of CuO additions on the density of KNN. The authors reported problems with the mechanical instability of the ceramics after contact with water once the CuO is added to the stoichiometric KNN. The ceramics with an A/B ratio lower than 1 formed a secondary phase determined as K_4_CuNb_8_O_23_. The authors suggested that Cu^2+^ acts as an acceptor dopant that goes to the B-site, and at the same time Cu^2+^ formed the secondary phase K_4_CuNb_8_O_23_ with a melting point below the sintering temperature, acting as a liquid sintering aid. Since the presence of the K_4_CuNb_8_O_23_ secondary phase was shown to improve the density, they added 0.5% of the synthesized K_4_CuNb_8_O_23_ phase to the stoichiometric KNN and achieved a density of 4.4 g/cm^3^ (the relative density was not given). The authors reported that both the CuO addition and an A/B ratio < 1 are required for an improvement in the sinterability.

#### 3.6.2. Solid Solutions KNN + ABO_3_

Additions of LiNbO_3_, LiTaO_3_, and LiSbO_3_ can lower the sintering temperature of KNN, but their influences on the grain size distribution differ [14,136,137,138,139]. Wang *et al*., prepared LiNbO_3_-modified KNN ceramics at a temperature as low as 950 °C with the aid of excess Na_2_O, where a significantly increased grain size was observed [137]. In contrast, Ta and Sb dopants are beneficial for reducing the grain size and achieving a uniform grain-size distribution. However, the solid solutions between KNN and BaTiO_3_, CaTiO_3_, CaZrO_3_ need higher sintering temperatures, normally around 1200 °C [19,64,140]. In addition, it was revealed that both BaTiO_3_ and CaZrO_3_ can induce abnormal grain growth behavior in KNN ceramics [19,64].

### 3.7. Alternatives to Conventional Sintering Methods

Beyond chemical modifications, special sintering techniques can be employed to further enhance the performance of piezoelectrics. Among the oldest of these techniques is pressure-assisted sintering (PAS), whereby the application of external, uniaxial (hot forging) or isostatic (hot-isostatic pressing) pressure provides an additional driving force for sintering and so significantly enhances the densification rate relative to the coarsening rate [40,41]. The benefit of this method for the sintering of KNN was demonstrated as early as the 1960s, yielding ceramics with relative densities exceeding 99% and the highest electromechanical properties reported for pure KNN [141,142,143]. In addition, ceramic samples with relative densities close to the theoretical value could be achieved for the end-members of the KNN system, *i.e.*, KNbO_3_ [144] and NaNbO_3_ [90].

Spark-plasma sintering (SPS) is, in principle, similar to PAS, but it possesses some unique features, e.g., a low sintering temperature, a short soaking duration, a large current shock effect, *etc.* [145,146]. For instance, pure KNN ceramics have been successfully sintered at a temperature as low as 920 °C, which is about 200 °C lower than that for conventional sintering [59]. Relative densities of up to 99% were achieved and the *d*_33_ was increased up to 148 pC/N. Additionally, KNN-based ceramics prepared using SPS show a superior fracture strength to conventionally sintered ceramics, benefiting from the improved densification and fine-grained microstructures [147].

Another recently emerging alternative to conventional sintering is microwave sintering (MWS), whereby the heating is achieved by the volumetric absorption of electromagnetic energy. This results in homogeneous heating, enables high heating rates, enhances diffusion processes, and reduces energy consumption [148,149]. Feizpour *et al.* reported the use of MWS to sinter pure KNN [150]. Although the obtained density and the electromechanical properties were comparable to conventionally sintered samples, the process time was decreased and the grain size was reduced from 6.6 to 3.8 μm. A more evident benefit of MWS was reported for the (1–*x*)(K_0.48_Na_0.48_Li_0.04_)(Nb_0.96_Ta_0.04_)O_3_-*x*SrTiO_3_ by Bafandeh *et al.* [151]. Samples with a relative density of 99% and a fine-grained microstructure were obtained at an approximately 100–200 °C lower sintering temperature and with a shorter sintering time, as compared to conventional sintering. The improved electromechanical properties were attributed to the increased density and the smaller losses of volatile alkalis.

However, the above-mentioned sintering techniques (PAS, SPS, MWS) come with some limitations, which today still prevent their widespread industrial application and restrict their use to laboratories. Among the most significant are the high costs of the equipment and, in the case of commonly used carbon dies, the reduction of the oxide ceramic sample. The latter can be partially compensated by post-annealing in air; however, the influence of the reduction and the re-oxidation on the sample’s defect state remains as an open question.

An alternative way to improve the sintering is to modify the firing schedule, either by using fast firing [152], rate-controlled sintering [153], or two-stage sintering [154]. Most of these techniques exploit the fact that different sintering mechanisms are activated in different temperature ranges and aim at enhancing the densification mechanisms, while preventing grain growth. However, the application of these techniques to the pure KNN system and other alkaline niobates did not result in an improved density and finer microstructure, although some improvement was demonstrated for doped KNN systems [155]. This could be related to the low activation energy of the surface diffusion (Section 3.3.1 and Ref. [90]), which becomes activated even at high heating rates, coarsens the microstructure and reduces the driving force for densification.

### 3.8. Solid-State Crystal Growth

Solid-state crystal growth (SSCG) is a special method for growing single crystals based on the phenomenon of abnormal grain growth in a polycrystalline matrix [156]. In this method, a single crystal (the seed crystal) is buried in a ceramic powder, pressed into a pellet and then sintered. The single crystal of the ceramic composition then grows on the seed crystal. Alternatively, the seed crystal may also be placed on top of a pre-sintered and polished ceramic specimen [157]. The SSCG method has the following advantages over traditional crystal growth methods from the vapor or liquid phase: conventional furnaces can be used, expensive Pt crucibles are not required, compositional variation or solute segregation in the crystal is minimized and temperature-gradient-induced stresses are avoided. However, it is difficult to grow large single crystals using this method, as large seed crystals are required.

In order to improve the piezoelectric properties of KNN-based ceramics, much work has been carried out on single-crystal growth, e.g., Refs. [158,159,160,161]. The SSCG method has been used to grow single crystals of KNN [162], K_0.5_Na_0.5_NbO_3_-LiTaO_3_ [163] and K_0.5_Na_0.5_NbO_3_-SrTiO_3_ [164].

**Figure 14:**
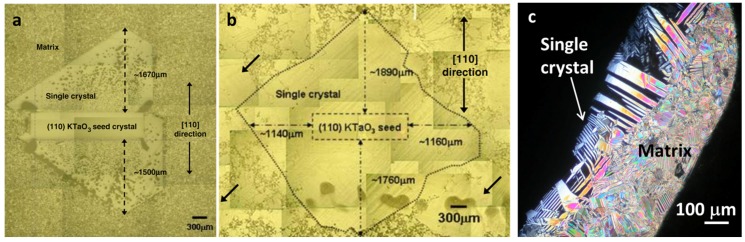
Optical micrograph of the KNN single crystal grown by solid-state crystal growth in a conventional furnace (**a**) and grown in a hot press; arrows indicate the abnormal grains in the matrix (**b**) (Reprinted with permission from [165]. Copyright 2008 The American Ceramic Society). (**c**) Domain structure of KNN single crystal under polarized optical microscope [Courtesy of Elena Tchernychova].

During atmospheric sintering the porosity in the polycrystalline KNN matrix can become trapped in the single crystal (Figure 14a). The porosity in the single crystal can be reduced by hot-pressing the sample before crystal growth [162] or by growing the crystal in a hot press [165]. During annealing in a hot press at 1100 °C and 50 MPa for 100 h, a single crystal up to 4 mm in diameter was grown on the KTaO_3_ seed (Figure 14b). The obtained single crystal with a monoclinic symmetry was composed of large, 90° domains of up to 100 μm in width, which additionally consisted of smaller 180° domains with widths from 50 to 300 nm (Figure 14c), and with the exact K_0.5_Na_0.5_NbO_3_ chemical composition [166]. This single crystal exhibited ferroelectric, dielectric and piezoelectric properties comparable to the properties of the KNN ceramic and showed an extremely high eletrostrictive response (*M_33_* = 2.59 × 10^−14^ m^2^/V^2^, measured with an atomic force microscopy at 2 Hz), which was explained by the extrinsic strain from the 180° domain-wall motion [167].

### 3.9. KNN Versus PZT: Sintering and Vapour Pressure

Whenever the piezoelectric properties of lead-free piezoelectrics are discussed, they are compared to those of lead-based piezoelectrics, mainly with their most widely used representative PZT, see for example [6,8].

**Figure 15:**
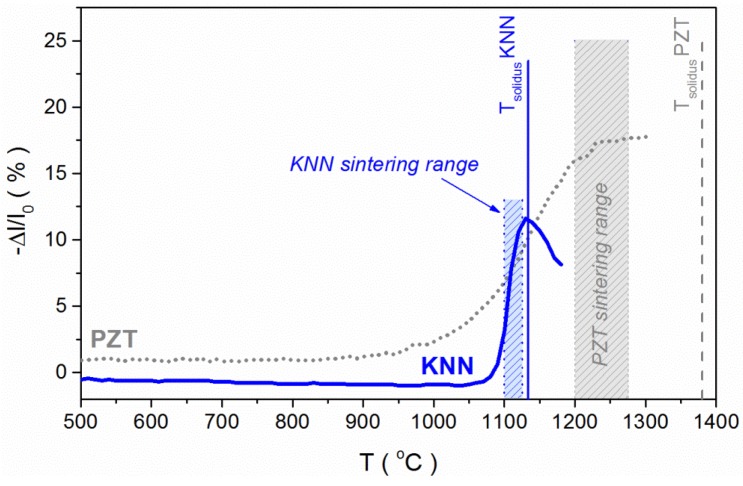
Shrinkage *versus* temperature curves of KNN and Pb(Zr_0.53_Ti_0.47_)_0.98_Nb_0.02_O_3_ (PZT) powder compacts with the commonly employed sintering temperature ranges. The solidus temperatures of both materials are indicated [25]. The KNN powder was prepared from the mixture of alkali carbonate and niobium oxide powders with two calcinations at 800 °C for 4 h and intermediate milling. The PZT powder was synthesized by a double calcination of a homogenized oxide-powder mixture at 900 °C for 4 h with intermediate milling. The sintering curves of both materials are from the authors' archive.

If we compare the sintering curves of KNN and PZT (donor-doped morphotropic-phase-boundary composition) (Figure 15), it is evident that the shrinkage interval of KNN, as previously discussed in Section 3.3, is indeed much narrower than that of PZT: even less than 100 °C [91], in contrast to a few 100 °C for the latter [38,39,168]. The sintering temperature range of KNN is just below its solidus temperature, which is not the case for PZT, meaning that from the experimental standpoint the sintering of KNN requires much more precise temperature control than that for PZT. Furthermore, as also discussed in Section 3.3, the activation of the surface diffusion in the initial sintering stage, which contributes to the coarsening of the microstructure, hinders the densification of KNN, while reaching high density for the PZT is not a problem.

The next critical point of comparison for the KNN and the PZT is the high vapor pressure of the alkalis over the KNN at the processing temperatures. The high vapor pressure of lead oxide over PZT during sintering [36,37,169] can be effectively controlled by the proper choice of atmosphere powder [38,39]. Furthermore, the perovskite phase is stable, even with a certain level of lead sub-stoichiometry [37,170]. Note that neither the level of the alkali sub-stoichiometry of the (K,Na)NbO_3_ solid solution nor the phase relations in the ternary K_2_O-Na_2_O-Nb_2_O_5_ system are known, although ternary phases are expected on the basis of the phases existing in the K_2_O-Nb_2_O_5_ and Na_2_O-Nb_2_O_5_ systems, see for example [76,77,78,79].

**Figure 16:**
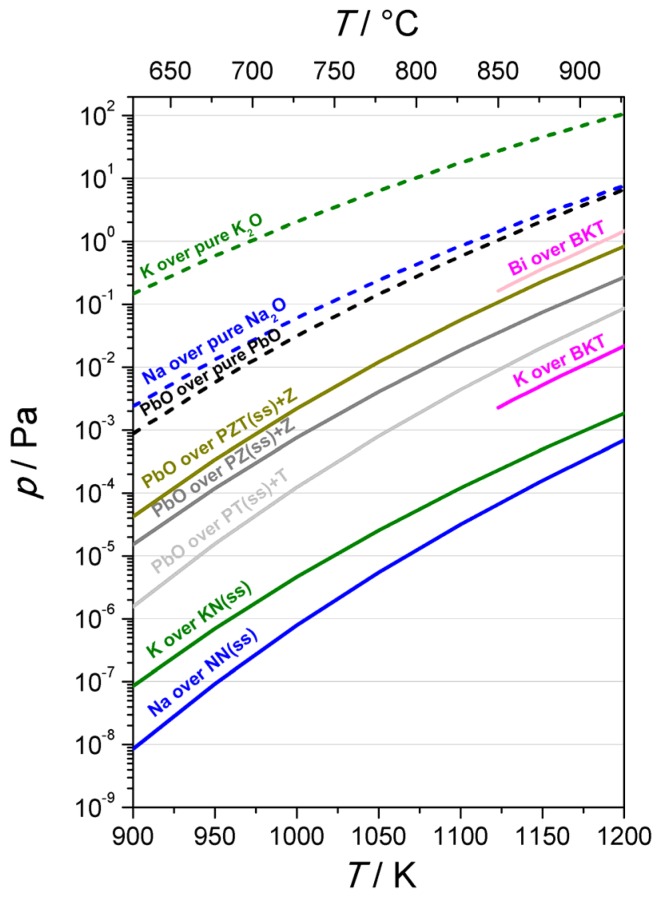
Vapor pressures of potassium [86] and sodium [172] over the respective oxides and niobates, of lead oxide over PbTiO_3_ + TiO_2_ (PT + T), PbZrO_3_ + ZrO_2_ (PZ + Z), Pb(Zr_0.5_Ti_0.5_)O_3_ + ZrO_2_ (PZTss + Z) [36,173], and of bismuth and potassium over Bi_0.5_K_0.5_TiO_3_ (BKT) [171]. Note that in the calculation of the vapor pressure of lead oxide and lead-based perovskites, denoted as “PbO” in the graph, the contributions of monomeric lead oxide (PbO), and also of atomic species (Pb) and dimeric oxide (Pb_2_O_2_) were taken into account [86,173,174]. (Reprinted with permission from [86]. Copyright 2015 The Royal Society of Chemistry).

In the frame of the Knudsen-effusion mass-spectrometry study of the KNbO_3_-NaNbO_3_ system, the vapor pressures of the alkalis over the respective alkali oxides and niobates (see Section 3.2) were compared with the vapor pressure of lead oxide over PbO and PZT in the temperature interval 627–927 °C, see Figure 16 [86]. Note that the main alkali species in the vapor phase in equilibrium with the condensed phase are alkali atoms and oxygen molecules. The vapor pressure of the potassium over potassium oxide is ~10^2^ Pa at 927 °C, which is more than one order of magnitude higher than that of sodium over sodium oxide. The vapor pressures of both alkalis over the respective niobates are more than five orders of magnitude lower, and the respective values of potassium and sodium at 927 °C are ~2 × 10^−3^ and ~6 × 10^−4^ Pa. The vapor pressures of both alkalis change only slightly within the whole compositional range of the solid solution.

Interestingly, the vapor pressure of lead oxide over PbO almost coincides with the data for the sodium vapor pressure over the pure oxide across the whole temperature range, but the vapor pressure of lead oxide over PZT + ZrO_2_ (~1 Pa at 927 °C) is almost three orders of magnitude higher than the respective values of the alkalis over the alkali niobates.

Figure 16 compares the above data with the vapor pressures of volatile species over Bi_0.5_K_0.5_TiO_3_ (BKT) [171], as a representative of another group of lead-free piezoelectric ceramics [8]. The vapor pressure of bismuth over BKT is very close to the value of lead oxide over PZT + ZrO_2_, while that of the potassium is about two orders of magnitude lower.

## 4. Conclusions

The sintering behavior of KNN ceramics, including the narrow sintering range, the poor densification, the appearance of secondary phases, and the inability to control the microstructure, has been a recurring processing problem for this lead-free piezoelectric. In this contribution, we have reviewed the current knowledge about the sintering of KNN.

The inherent properties of the reagents in the solid-state synthesis of KNN, including the strongly hygroscopic nature of potassium carbonate, the different reactivity and diffusion rate of the constituent ions, and also the different vapor pressures of the potassium and sodium species, indicate that obtaining a phase-pure, stoichiometric and chemically homogeneous perovskite ceramic powder requires extreme care, and, importantly, these are prerequisites for obtaining materials with reproducible microstructures and properties.

The study of the sintering mechanism during the initial stage, performed on the end-member of the KNN solid solution, NaNbO_3_, revealed that due to the low activation energy for the surface diffusion the latter mechanism contributed to extensive grain growth without densification in the initial sintering stage, and consequently resulted in a reduced driving force for further densification.

The sintering interval of the stoichiometric KNN is usually limited to a few 10 s of °C below the solidus temperature at 1140 °C. Introducing excess alkali to the KNN contributed to a down-shifting for the onset of shrinkage to 800 °C, but extensive coarsening of the microstructure hindered the densification. In contrast, excess niobium in the KNN slightly broadened and up-shifted the densification interval compared to the stoichiometric formulation and contributed to a finer microstructure, but also lower density. Sintering in the presence of a liquid phase, such as copper- or zinc-based, contributed to an effective densification, but not to a decrease in the sintering temperature, which was achieved when a borate- or germanate-based glassy phase was implemented. The chemical modification of the KNN via donor doping was expected to enhance the densification by the introduction of cation vacancies, which was indeed the case for low dopant concentrations. Acceptor doping (Ti, Zr) on the Nb-sites, resulting in the formation of vacancies in the oxygen sublattice, contributed to the enhanced densification, which was not expected on the basis of a comparison with other perovskite materials. In complex solid solutions of KNN with lithium niobate, tantalate, antimonate or with alkaline earth perovskites (CaTiO_3_, CaZrO_3_, BaTiO_3_), the sintering temperatures were changed as compared to KNN, and in some cases abnormal grain growth was observed. Sintering KNN in reducing conditions contributed to an enhanced densification and normal grain growth, in contrast to sintering in air or oxygen.

As a final note, the sinterability of KNN strongly depends on the stoichiometry, *i.e.*, the alkali/niobium molar ratio, and is thus very much dependent on the quality of the KNN powder. The sintering interval of stoichiometric KNN is just below the solidus temperature, and therefore the sintering of KNN requires much more care than the sintering of PZT. However, the vapor pressures of alkalis over their respective niobates are much lower than the vapor pressure of lead oxide over PZT (~10^−3^ Pa *versus* ~1 Pa at ~900 °C), meaning that the evaporation of alkalis during sintering is not as large as anticipated in earlier investigations and is not as difficult to control as in the case of PZT. The understanding of sintering for KNN and of different approaches to enhance its densification, discussed above, nevertheless provide the means for obtaining materials with good performance.
